# Advancing Glioblastoma Supportive Care: Closing the Caregiver Gap

**DOI:** 10.3390/cancers18213557

**Published:** 2026-11-05

**Authors:** Jordan L. Smith, Garin G. Griffith, Hala Hassanain, Jennie W. Taylor, Susan M. Chang, Kerri Winters-Stone, Ahmed Raslan, Stephen G. Bowden, Josh Walker, Ramon F. Barajas, Anh Huan Vo

**Affiliations:** 1 Department of Neurological Surgery, Oregon Health & Science University, Portland, OR 97239, USA; 2 Center for Experimental Therapeutics, Oregon Health & Science University, Portland, OR 97239, USA; 3 School of Medicine, Oregon Health & Science University, Portland, OR 97239, USA; 4 Department of Internal Medicine, Division of Hematology & Oncology, Oregon Health & Science University, Portland, OR 97239, USA; 5 Department of Neurology, University of California, San Francisco, San Francisco, CA 94143, USA; 6 Department of Neurological Surgery, University of California, San Francisco, CA 94158, USA; 7 UCSF Neuro-Oncology Gordon Murray Caregiver Program, San Francisco, CA 94143, USA; 8 Department of Oncological Sciences, Oregon Health & Science University, Portland, OR 97201, USA; 9 Translational Oncology Program, Knight Cancer Institute, Oregon Health & Science University, Portland, OR 97239, USA; 10 Department of Radiation Medicine, Oregon Health & Science University, Portland, OR 97239, USA; 11 Department of Cell, Developmental & Cancer Biology, Oregon Health & Science University, Portland, OR 97239, USA; 12 Neuroradiology, Department of Radiology, Oregon Health & Science University, Portland, OR 97239, USA; 13 Advanced Imaging Research Center, Oregon Health & Science University, Portland, OR 97239, USA; 14 Neuro-Oncology, Oregon Health & Science University, Portland, OR 97239, USA

**Keywords:** caregiver, dyadic intervention, exercise oncology, glioblastoma, high-grade glioma, palliative care, supportive care

## Abstract

Glioblastoma (GBM) is devastating both in prognosis and in patient impact. Differing from other solid tumors, GBM produces significant, often rapid, neurologic decline necessitating intense caregiving. This care usually falls to an intimate partner or family member. Early studies in GBM patient–caregiver dyads confirm substantial caregiver needs. Most caregivers report significant distress, with nearly 40% reporting depression and anxiety. Educational initiatives, early palliative care, and psychotherapy have increased caregiver preparedness. However, current healthcare delivery is organized primarily around the patient, and few systems offer caregiver-specific interventions. Early evidence suggests that focused efforts to improve caregiver quality of life may improve patient survival and healthcare utilization, incentivizing the healthcare team to further investigate how to best support the patient–caregiver dyad. The literature in other tumor types suggests dyad-based interventions may also reduce distress more effectively than caregiver or patient-based interventions alone. Further research is needed to design an optimal supportive-care model for GBM dyads. Across each stage of the disease course (from diagnosis and treatment to surveillance, progression, and bereavement), the needs of the dyad are dynamic. This requires targeted and adaptive interventions such as nurse-led navigation, structured education, anticipatory guidance, and clinical supportive-care programs or clinical trials aimed at improving dyadic physical and psychological well-being at different time points during the disease course. Ultimately, future studies evaluating caregiver support in GBM are needed and should assess not only psychological benefits and patient survival, but also programmatic implementation, clinical trial participation, and healthcare utilization efficiency.

## Simple Summary

This narrative review synthesizes the current literature to propose a supportive-care framework for dyadic interventions in patients with glioblastoma (GBM) and their caregivers. Based on a comprehensive database search, we examined key caregiver needs and evaluated various intervention modalities, including dedicated caregiver initiatives, educational and navigation programs, and clinical trials addressing dyadic physical and psychological well-being. Although further research and clinical trials are required, emerging evidence underscores caregiver support as a critical need in GBM supportive care.

## 1. Introduction

Despite decades of dedicated research, glioblastoma’s (GBM) prognosis remains devastating, with a median survival of less than 20 months [1]. A diagnosis of GBM often causes profound psychological and physical distress for patients and their caregivers [2]. Caregivers of patients with GBM are typically intimate partners or family members. Standard of care treatment for GBM is intensive for patients and caregivers alike. Typically, patients undergo upfront surgical resection, followed by concomitant fractionated radiation and chemotherapy, and ultimately chemotherapy alone [3]. Unlike many chemotherapy regimens that require intravenous administration in the clinic, patients with GBM receive an oral alkylating chemotherapy, temozolomide (TMZ). TMZ is administered at home under the direction of a caregiver [4,5]. Responsibility for at-home chemotherapy rapidly transforms the caregiver from spouse, sibling, or child to clinical provider: a challenging role to assume, and one most are not prepared for.

Neuro-oncology has long recognized the caregiver as more than a passive companion. To date, the literature exploring the caregiver–patient dyad in GBM is primarily observational; however, it does include several randomize control trials (RCTs), meta-analyses, and systematic reviews (Table 1). A larger body of evidence exists for patient–caregiver dyads in high-grade glial tumors (HGGs) and more broadly in patients with primary brain tumors (PBTs). While patients with GBMs share some clinical similarities to other patients with PBTs, the GBM patient–caregiver dyad experience presents unique and significant challenges secondary to GBM compressed disease course, and the rapid neurologic decline experienced by most patients. One notable limitation of the GBM caregiver literature is the lack of diversity in the study population. The studied caregiver cohort is predominantly white, female, and of higher socioeconomic status. In many studies, non-native English speakers, indigent and low socioeconomic status caregivers, and racial minorities are not well-represented, limiting the generalizability of the findings.

Within the constraints of the predominant study population, caregiver distress and poor quality of life (QoL) are exceedingly common in caregivers of patients with GBM. A 2026 meta-analysis of ten studies of glioma dyads estimated clinically significant caregiver burden in 60%, distress in 68%, depression in 35%, and anxiety in 36%. The study authors do advise caution in interpretation of results given the limited sample size, lack of pooled outcomes for many studies, and variable methodology across the included samples [6]. Across all PBTs, increased caregiver distress has been associated with younger caregiver age, increased financial stress, unmet caregiver needs, low levels of outside support, and reduced caregiver mastery [7,8,9]. Herein caregiver mastery will refer to the caregivers’ self-perception and confidence in their behavior throughout the caregiving process [10].

Many groups have recognized the need for caregiver-specific interventions, citing caregivers expressing support needs distinct from patient needs [11]. Existing interventional programs in GBM dyads have improved caregiver knowledge, preparedness, and mastery. Few have produced broad and durable reductions in caregiver distress, burden, and QoL (Table 1) [8,12,13,14,15,16,17,18].

**Table 1:** Selected completed supportive-care interventions and implementation models for high-grade glioma caregivers and patient–caregiver dyads.

Study	Population Design	Intervention	Delivery	Comparator	Outcomes	Main Findings	Key Limitation
Boele et al. [15]	56 HGG patient–caregiver dyads; randomized controlled trial	Caregiver/dyad; psychoeducation and cognitive-behavioral strategies	Six 1 h psychologist sessions; follow-up to 8 months	Care as usual	Mental HRQOL; caregiver mastery	Intervention maintained caregiver mental HRQOL and improved mastery	Small sample; broad HGG population; limited implementation data
Halkett et al. (Care-IS) [16]	188 HGG caregivers during chemoradiation; randomized controlled trial	Caregiver; needs assessment, tailored resource folder, home visit, monthly support	Nurse-led; up to 12 months	Usual care	Preparedness, distress, anxiety, depression, QOL, competence, strain	Preparedness improved through 12 months; distress and secondary outcomes did not	Preparedness benefit did not generalize to emotional outcomes
Applebaum et al. [12]	60 GBM caregivers; pilot randomized controlled trial	Caregiver; existential distress, meaning, identity, agency	Seven individual psychotherapy sessions	Enhanced usual care	Personal meaning, benefit finding, depression, spiritual well-being	Improved meaning and multiple secondary psychological outcomes	Pilot size; short follow-up; limited demographic diversity
Golla et al. (EPCOG) [14]	217 patients with newly diagnosed or recurrent GBM plus caregivers; phase III randomized trial	Primarily patient/family; early integrated palliative care	Monthly palliative care plus optimized standard care for 12 months	Optimized standard care	Caregiver burden; patient QOL, mood, palliative-care problems	Patient outcomes improved in selected analyses; caregivers did not appear to benefit	Survival imbalance complicates QOL interpretation; caregiver intervention content limited
Leo et al. (EMPOWER GBM) [13]	14 interview participants; 11-person single-arm pilot	Patient, caregiver, or dyad; symptom management, autonomy, caregiving efficacy, connection	Six flexible sessions	None	Psychological outcomes, functional well-being, caregiving efficacy, acceptability	Feasible and acceptable with favorable pre-post signals	Very small uncontrolled pilot
Milbury et al. [19]	20 glioma dyads during radiotherapy; pilot randomized trial	Dyad; breathing, gentle movement, guided meditation/yoga	Twelve sessions during radiotherapy	Waitlist control	Caregiver depression, fatigue, mental QOL; patient symptoms and QOL	Feasible; clinically meaningful effect estimates for patient and caregiver outcomes	Very small sample; preliminary efficacy only
Milbury et al. [20]	67 glioma dyads during radiotherapy; three-arm pilot randomized trial	Dyadic yoga versus caregiver-only yoga	Fifteen sessions; in person or videoconference	Usual care	Mental QOL, caregiving reactions, subjective benefit	Caregiver-only yoga produced greater subjective benefit and favorable effect estimates on several outcomes	Pilot power; sample predominantly female and non-Hispanic White
Page and Chang [21]	Neuro-oncology caregiver program; practice/implementation report	Caregiver; education, support, navigation, and program development	Clinic-integrated program	Not applicable	Program reach, identified caregiver needs, services delivered, bereavement contacts	Provides an implementation model for recognizing caregivers within neuro-oncology	Not an efficacy trial

**Figure 1:**
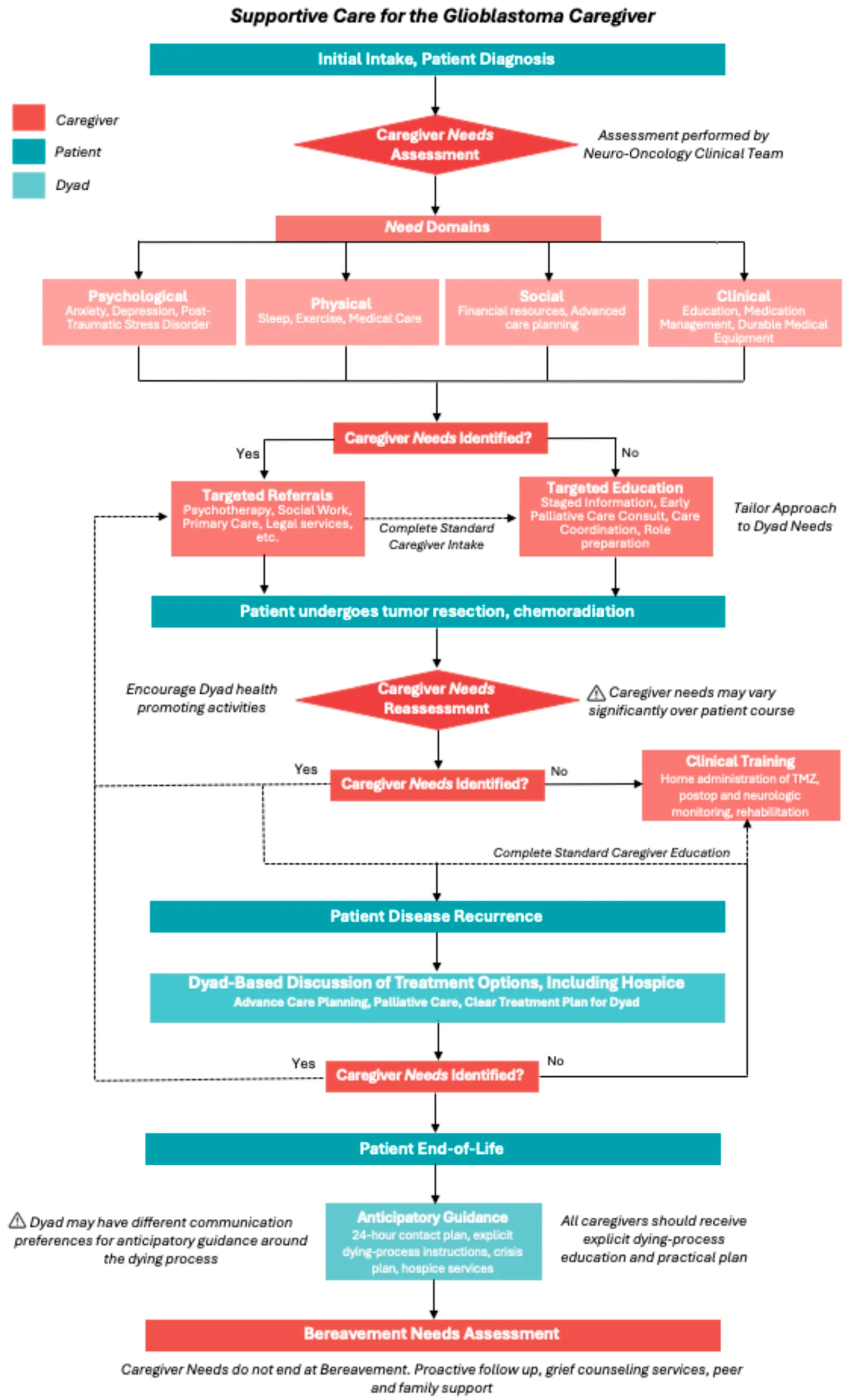
Assessing and addressing supportive care for the glioblastoma caregiver.

This review synthesizes caregiver needs across the GBM disease course critically evaluating completed supportive-care interventions (Table 1 and Table 2). In addition to the existing trialed interventions, we propose consideration of a strategy that has shown promise in other tumor types: structured dyad exercise. Structured exercise interventions have shown promise in solid tumor dyads; however, the limited intervention-based literature exists for GBM. We propose that caregiver preparedness and education are necessary, but insufficient, to promote caregiver well-being. Supportive care for the dyad should aim to preserve both patient and caregiver physical and psychological health. Whether these interventions improve patient survival remains unknown, although observational studies have identified an association between caregiver mastery and GBM patient survival (Figure 1) [22,23].

## 2. Materials and Methods

This structured narrative review was informed by a focused literature search conducted from 31 July through 6 August 2026. PubMed/MEDLINE, Embase, PsycINFO, and CINAHL were searched using combinations of terms related to glioblastoma, high-grade glioma, primary brain tumors, caregivers or care partners, caregiver burden, unmet needs, supportive and palliative care, dyadic interventions, exercise, physical activity, rehabilitation, and bereavement. *Claude Opus 4.8* (*Anthropic; released 28 May 2026*) and *Consensus 2.0*, an AI-powered academic search engine, were used to scope preliminary search terms for the review. All search queries and frameworks produced in conjunction with these tools were used only for initial orientation. All identified articles were retrieved and reviewed by the study authors. Articles were included only after our critical evaluation. Reference lists of relevant systematic scoping reviews and key primary studies were also reviewed to identify the additional literature. Completed peer-reviewed studies were prioritized; conference abstracts were excluded. Evidence from other cancer populations was included when direct neuro-oncology evidence was limited and the external literature informed intervention rationale or design.

This is a narrative review rather than a systematic review. It was designed to integrate clinically adjacent studies and develop a care framework, not to provide an exhaustive study census or pooled treatment effect. Formal duplicate screening, risk-of-bias adjudication, and meta-analysis were therefore not performed. Claims are calibrated to study design: feasibility is distinguished from efficacy, observational associations are not interpreted as causal effects, and patient benefit is not assumed to imply caregiver benefit.

The authors retain complete responsibility for the scope of the article and integrity of the literature search. The authors are responsible for complete generation of the manuscript, table and figure contents.

## 3. Why Caregiving in GBM Is Distinct

Caregiving for patients with GBM is complex, combining the practical responsibilities of advanced cancer care with the consequences of progressive neurologic disease [6,24]. Unlike other solid tumor types, e.g., breast, lung, liver, etc., patients with GBM often experience early, significant neurologic morbidity secondary to tumor progression or as a consequence of intracranial radiation. The rapid disease course permanently alters patient and caregiver lifestyle, allowing very little time for dyads to adapt to the new normal. Patients with GBM frequently experience altered cognition, language, executive function, mobility, and decision-making capacity. Further, neurologic symptoms may fluctuate, complicating the ability of a caregiver to assess the patients’ needs. Given the progressive and unpredictable symptoms patients with GBM experience, completion of activities of daily living (ADLs) and cohesive oncologic care often rely on the intensive assistance of a caregiver. The caregiver typically manages medications, monitors for concerning neurologic conditions including seizure or hydrocephalus, coordinates appointments, and provides transportation and personal care. As the cognitive consequences of the disease progress, the caregiver may be required to assume all clinical decision-making on behalf of the patient, further increasing feelings of despair or distress (Table 2).

Several studies support the close relationship between poor patient function and caregiver distress. A multicenter study, including seven academic centers, recruited 167 GBM dyads to undergo an assessment of caregiver burden and QoL. At the time of administration, the survey had not been validated. The caregivers recruited were predominantly white, female, college-educated and with household incomes >$100,000. The study authors report that poor patient performance status was associated with more caregiving tasks and overall greater caregiving time. The authors hypothesized patient-specific disease characteristics directly impacted caregiver QoL. They found patient confusion was negatively associated with all reported caregiver “needs domains” including emotional and social health, general wellness, occupational stability, financial comfort, and overall QoL domains [41].

Nearly every domain of life for GBM dyads changes rapidly after initial diagnosis. Dyads must rapidly adopt new coping strategies to maintain their emotional, physical and financial health. The strategies patients with PBTs use to cope may also influence caregiver-perceived QoL. In a single-center prospective longitudinal study in France, 38 HGG dyads were recruited prior to surgical resection to evaluate coping strategies and QoL. The baseline assessment was performed 2–6 weeks post-resection, and the follow-up assessment at three months. The authors found that caregiver- and patient-coping strategies influenced caregiver QoL. Using validated self-reported metrics, the authors found that employing “social support” coping strategies improved caregiver QoL at three months after initial diagnosis but interestingly was associated with poorer QoL for patients. Further, “problem-solving” and “positive thinking” improved QoL for caregivers but were not related to QoL for patients. Similar to other caregiver studies, many dyads who were approached to participate declined the opportunity. The authors suggest caregiver burden may subsequently be underestimated. They hypothesize that the population least interested in clinical research participation may also represent the most burdened caregivers [42].

The rapid clinical course of GBM compared to other tumor types requires the caregiver to adapt to their role frequently and quickly. Making matters more difficult, most GBM caregivers are without formal training. Despite the lack of education, caregivers are expected to administer home postoperative care and home chemotherapy and reliably assess neurologic decline. The rapid transition and evolving identity from loved one to caregiver can occur immediately following diagnosis. Caregiver identity continues to transition through disease progression, end-of-life care, and bereavement. As disease progresses, caregivers often describe feeling less like a spouse, child, sibling, or friend, and more like a medication manager, safety monitor, healthcare navigator, and surrogate decision-maker (Table 1) [7,16,24,25,41,43,44,45]. A qualitative meta-synthesis of 16 studies across English and Chinese databases found that for caregivers of glioma patients role transition and continued education/informational support were central caregiver needs across the entire disease trajectory. A large proportion of the research was conducted in a distinct cultural population; as such, the findings may not be generalizable [25].

While several studies have described differing and sometimes opposing needs among patients and caregivers, a broader qualitative systematic review found dyads do experience some commonality along the disease trajectory. Specifically, a systematic review of PBT dyads that included 33 total studies and 671 participants (303 patients, 368 caregivers) found that patients and caregivers alike described loneliness, role renegotiation, fragmented services, and a need for coordinated, individualized support [26]. Taken together, these studies suggest that the needs of the GBM dyad are complex. While at times dyad needs are in conflict, ultimately both parties experience significant changes in QoL that require an iterative, multidisciplinary approach. The iterative approach required to identify and address caregiver support needs is detailed in Figure 1.

## 4. Caregiver Burden and Unmet Needs Across the Disease Trajectory

### 4.1. Diagnosis, Surgery, and Discharge

The caregiver role is not static. As patient needs change with surgical care, disease progression and ultimately end-of-life, caregiver support needs change rapidly in tandem. Herein, we organize caregiver needs around patient phase-of-care to best discuss temporally based supports and interventions (Table 2). Further, in Figure 1, we detail the iterative process to identify dyad support needs at each point in the disease course and appropriate next steps.

The initial period following diagnosis of GBM combines prognostic shock and an abrupt transfer of responsibility from patient to caregiver. Dyads experience significant disruption at work and in social and family roles, including care of children. Following diagnosis, most patients are admitted and undergo surgery quickly. There is little time for absorption of complex information in the acute phase. A safe discharge from the hospital post-surgery requires extensive involvement from the caregiver and social work. Many post-surgical GBM patients require acute or outpatient rehabilitation and installation of home health equipment. During this initial phase, anticipatory grief is common among patients and caregivers alike. Most caregivers leave the hospital with many new responsibilities, including medication management, wound monitoring, rehabilitation appointments, and recognition of urgent symptoms requiring emergency care. Patients may also experience new cognitive or neurologic deficits in the post-operative period, complicating their ability to communicate with their caregiver. A common source of caregiver distress is the inability to understand the patient’s needs.

Patients with GBM can decompensate rapidly. Caregivers frequently express feeling unprepared to recognize a patient’s clinical decline. The responsibility of making critical decisions with limited knowledge is a common source of hypervigilance and anxiety in caregivers. As discussed throughout this review, a central need of most caregivers is the desire for better education and information. Caregivers across the entire disease trajectory express the need for continued, targeted education. In a single-center longitudinal study, 25 family caregivers of PBT patients identified at office visits were interviewed about their decision-making, daily tasks and support needs over the course of their loved one’s disease. Notably, caregivers of low socioeconomic status, non-native English speakers, and members of racial and ethnic minorities were not represented. Nearly all of the included caregivers expressed feeling continually undertrained to make time-sensitive clinical decisions. Their findings are perhaps best summarized by one caregiver’s sentiment, “When one family member is ill, all of the family is ill.” [24].

The literature recommends that in the acute phase information be staged, repeated, and paired with practical demonstration rather than delivered as a single large packet [8,24,25,26]. A structured intervention found caregiver knowledge base could be improved with targeted education. Twenty-four caregivers of PBT patients underwent a multiple-choice test developed by expert consensus on brain tumor biology, care basics, and symptom management. The test was administered before, immediately following, and 4–6 weeks after the educational program. Caregivers scored significantly better immediately following education and at the 4–6-week mark compared to baseline. Authors note that while 4–6-week scores were better than baseline, they were significantly decreased compared to scores obtained immediately post-education, suggesting attrition of knowledge with time. Notably, the population for this study was predominantly white, female, spousal caregivers and did not include non-English speakers, limiting its broad applicability. Within this study population, the authors conclude that the knowledge drop-off highlights the need for continued, structured, and repeated education [8].

### 4.2. Chemoradiation and Early Outpatient Care

Chemoradiation typically follows surgery for most patients with GBM. During concurrent treatment, caregivers coordinate frequent visits for radiation and manage oral chemotherapy at home. Caregivers may also face additional psychological stress when considering clinical trial participation at initial diagnosis. Transitioning to chemoradiation and outpatient care is a major transition point and is often accompanied by caregivers expressing feelings of overwhelm (Table 2). In a single-center prospective cross-sectional study in the UK, structured interviews were completed with 19 patients with GBM, and 12 caregivers assessing their perspectives on treatment side-effects, and information provided prior to starting chemoradiation. Dyads expressed significant overwhelm by the volume of generalized information and disappointment in the lack of specific information on expected, probable toxicities of chemoradiation. Most dyads expressed a discrepancy between the information provided and the actual experience of chemoradiation toxicities. The authors conclude that most information provided at the initiation of treatment does not prepare dyads for the severity of toxicities, nor for the possibility that toxicities will fluctuate over treatment course. This work highlights a central tension in caregiver education: caregivers desire clear, realistic, detailed information, but simultaneously do not want to be overwhelmed with anticipatory guidance [27].

Discussed in greater detail in Section 5.1, Care-IS was an RCT that enrolled 188 caregivers of HGG patients to undergo targeted interventions to improve feelings of preparedness and to reduce caregiver distress while their loved ones were undergoing initial chemoradiation (Table 1). Targeted education did improve preparedness but did not improve feelings of distress [16]. While effective for improving caregiver’s sense of preparedness, educational initiatives alone may not meaningfully reduce caregiver distress in part because increased understanding does not reduce caregiver burden.

Many caregivers continue working while their loved ones undergo treatment and carry a significant daily task load. Economic constraints necessitate that many caregivers continue employment, intensifying burden. A meta-analysis of 12 studies with 143,303 total patients with GBM correlated lower economic income with poorer survival. The analysis did have several limitations, including selection bias and a lack of key variables considered, including insurance status [28].

Task load is not the only contributor to caregiver burden. Many caregivers identify anxiety as a major contributor to increased strain and reduced QoL. To better understand contributors to HGG caregiver anxiety, semi-structured interviews were conducted with 21 caregivers within 1–3 months of the patient’s death. The following six overall themes were identified: reassurance-seeking or avoidance, uncertainty, changing relationships, inadequate social support, devaluation of self-care, and difficulty communicating with clinicians. These themes highlight the complex nature of caregiver anxiety and the need for specific-targeted interventions. In summary, early outpatient care requires intensive continued education for the caregiver, increased daily task burden, worsening distress/anxiety and new challenges in balancing dual, competing identities as caregiver and family member. These themes highlight the complex nature of caregiver anxiety and the need for specific-targeted interventions.

### 4.3. Surveillance and Relative Stability

A period of radiographic stability may limit appointment volume and daily tasks but does not eliminate caregiver burden. Persistent neurologic deficits, scan-related anxiety, fear of recurrence, and significantly altered family dynamics contribute to continued caregiver distress during this phase of care (Table 2). Unique to neuro-oncology, patients face both cognitive dysfunction and emotional distress from diagnosis throughout their disease course. These overlapping needs support consideration of both patient and caregiver assessments throughout the disease trajectory [30,31]. Patient psychosocial distress over cognitive changes, loss of neurologic function, and prognosis can be challenging to navigate for caregivers. In a secondary analysis of a single-center, prospective observational study of 58 malignant PBT dyads in Germany, the study authors found that psychosocial distress experienced by patients at diagnosis significantly predicted caregiver distress, anxiety and depression over the disease course. The authors conclude that routinely identifying patients in distress may help identify at-risk caregivers. Several important caveats of this study that may impact its generalizability to patients with GBM include high attrition of study participants and the heterogeneity of study participants including patients with cerebral metastases [32].

Patients and caregivers alike experience existential distress as the patient begins to suffer from cognitive decline, personality changes, and neurologic deficit. A cross-sectional study using telephone interviews in glioma dyads examined the daily experience for patients and caregivers as patients experienced progressive cognitive decline. Reflexive thematic analysis found worry about cognitive decline was at first secondary to survival, and true concern only began to emerge after the initial shock of diagnosis, surgery and chemoradiation had passed. Further, the authors found that cognition-related resources, while available, were difficult to identify and access for caregivers [33].

Finally, with the immediate crisis of diagnosis passed, community support typically falls away, further stressing the limits of caregivers. A qualitative thematic study recruited female spousal caregivers through a secret Facebook group to write 500-word letters about their experience. A total of 101 letters were collected. The letters emphasize that caregiver needs fluctuate and then peak during progression, when many social supports have regressed [34].

### 4.4. Progression and Increasing Dependence

Disease recurrence commonly produces another rapid reorganization of responsibilities. Treatment decisions may involve uncertain benefits. Decisions about clinical trial participation at recurrence can add another layer of uncertainty and stress. In the setting of recurrence, there is no standard of care for patients with GBM [2,46].

The lack of evidence surrounding appropriate treatment at disease recurrence can produce significant anxiety in caregivers and patients alike. In a qualitative, prospective study of 15 adult patients with GBM and 13 caregivers at Leeds Teaching Hospital UK, semi-structured interviews found that caregivers and patients expressed the need for clear communication around treatment choices. The study population included both male (33.3%) and female caregivers (66.6%). Included patients had variable treatments at the time of enrollment including biopsy only through multiple lines of chemoradiation. Dyads expressed that they often understood proceeding with additional care was “the only option,” highlighting the need for frank discussions around all choices, including hospice, after disease progression [47]. Similarly, through semi-structured interviews completed with 19 former and current caregivers of patients with GBM, study authors found that caregivers most often described feeling unprepared, having limited time with clinicians, underusing rehabilitation, and remaining confused about hospice and palliative care. Again, the predominant study population was white, female spouses, potentially limiting generalizability [35].

Caregiver mastery, or the feeling of assuredness and control, can falter during progression. While critical for the health of the caregiver, caregiver mastery has also been shown to correlate with patient survival. In 88 GBM dyads, each one-point increase in caregiver mastery was associated with a 16.1% lower adjusted hazard of patient death [22]. The critical role of caregiver mastery in patient survival was again supported in a longitudinal study of 41 patient dyads [23]. Both studies are discussed in further detail in Section 5.2.

During periods of clinical transition, feelings of caregiver mastery may be improved by trust of the care team and an explicit discussion of treatment burden and support services [15,22,23,48,49]. In HGG patients across the Netherlands, 56 dyads were randomized to protocolized sessions with a psychologist to improve mastery or the standard of care. Importantly, dyads were excluded if patients had a life expectancy of less than three months. Participants skewed towards well-educated, female caregivers. The authors found that caregivers who underwent the targeted psychotherapy sessions reported a small improvement in feelings of mastery compared to the control group. The authors also found that caregivers’ mental health was susceptible to perceived changes in the patient’s neurologic health, irrespective of the intervention (Table 1) [15]. Whether targeted psychotherapy will reliably improve caregiver mastery during disease progression requires further study.

Psychological support, even when caregiver-directed, is often experienced as patient-centered. In Australia, 11 current and eight bereaved caregivers of patients with HGG were recruited and underwent semi-structured qualitative interviews with reflexive thematic analysis. Most participants were female spouses. Education, race, and socioeconomic status were not reported. Several themes were identified in the interview including caregivers suppressing their own needs because, in their words, care was “never about me” [36].

### 4.5. End of Life and Bereavement

Symptoms can progress rapidly near the end, further challenging communication between caregivers and patients (Figure 1, Table 2). Progressive somnolence, dysphagia, seizures, agitation, weakness, and loss of communication can make the terminal phase particularly demanding. In a retrospective study completed across two neurosurgical departments in Italy, 45 caregivers of patients with GBM were screened with a validated end-of-life questionnaire which examined the caregiver’s view of the patient’s terminal phase. One limitation of the study is that approximately 43% of caregivers approached declined to be in the study, likely secondary to the sensitivity and distress of the subject matter. The authors suggest that caregivers who did enroll likely skew towards higher baseline caregiver mastery and may not represent the broader population. Unlike other studies discussed herein, the caregiver population in this study was predominantly of “low grade” education, and 30% were male. In this study, caregivers identified patient fatigue, reduced awareness and patient sadness as major drivers of caregiver distress and low QoL. Further, caregivers identified burnout and inadequate information as additional drivers of significant distress during end-of-life care [39].

Feeling underprepared for imminent death of a loved one is another driver of caregiver distress. A prospective study enrolled 55 family caregivers of HGG within approximately one month of the patient’s death to participate in a survey; they were very confident about what to expect during the dying process, while 72.5% felt very confident about what to do at the time of death. Additionally, 46% reported lacking a healthcare team member to turn to in distress [38].

Following the death of a loved one, caregivers often report complex and varied emotions. Many display symptoms consistent with anxiety, depression, and post-traumatic stress disorder. A mixed-methods cross-sectional study recruited 105 caregivers of patients with brain tumors who had been bereaved for at least six months, primarily through social media and charitable organizations. Among 81 caregivers who had been bereaved for at least 12 months, 64.2% (52/81) screened positive for prolonged grief disorder. In the broader cohort, 41.9% screened positive for post-traumatic stress, while approximately 35% reported depressive symptoms and 61% anxiety symptoms [40]. The authors stress the importance of timely interventions in bereaved caregivers to prevent psychological morbidity. The authors stress the importance of timely interventions in bereaved caregivers to prevent psychological morbidity.

## 5. What Existing Supportive-Care Interventions Have Accomplished

Most interventions tested neuro-oncologic dyads which involve psychoeducation, psychotherapy, care coordination, and early-phase dyadic programs. In GBM specifically, very few prospective interventional randomized trials exist examining the caregiver–patient dyad. Most of the trials remain small in part due to the rarity of the disease. To increase recruitment, many studies expand beyond GBM to include HGG tumor patient dyads, or PBT patient dyads. Most brain tumor types vary significantly in disease course and required treatment; however, given the relative paucity of evidence in the field, many have appropriately justified studying the larger populations. Further complicating interpretation, most of these prospective studies use different outcomes and test interventions with different targets. With consideration for the varied and limited evidence, the literature suggests that early education and navigation is valued by caregivers but does not reduce distress. Psychotherapy and psychoeducation may be beneficial to reduce caregiver distress. Finally, the role of early palliative care in benefiting caregiver well-being warrants further study.

### 5.1. Education and Navigation

Educational interventions have addressed a clear need expressed by caregivers, but knowledge and emotional health are not interchangeable. The largest completed HGG caregiver RCT interventional trial, Care-IS, randomized 188 Australian caregivers of patients with HGG across eight sites to a nurse-led program comprising an unmet-needs assessment, tailored resource folder, home visit, and monthly telephone support or usual care (Table 1). Primary outcomes were caregiver preparedness and distress at 2, 4, 6 and 12 months. Secondary outcomes assessed included psychological outcomes including anxiety and depression, QoL, competence of the caregiver, and overall QoL. Participants were recruited during chemoradiation. To be eligible for the study, patients had to be within two months of diagnosis, >18 years old, and English-speaking. Female, married spouses made up the majority of caregivers in the study. The authors found that preparedness improved at four months and remained high through 12 months. Distress, anxiety, depression, quality of life, competence, and strain did not significantly differ between groups [16]. This work suggests that a caregiver can become better prepared to perform difficult work, while remaining distressed by the work itself. One practical way to identify the needs of a caregiver of a patient with GBM in the clinical setting (Figure 1) is the six-item SCNS-P&C-6 from Care-IS. The brief tool captured cancer-impact and information/communication needs. The tool missed relatively few caregivers with needs on the full instrument. This work suggests screening should be linked to clear referral pathways [50].

A unique, dedicated caregiver program in neuro-oncology at the University of San Francisco demonstrated clinical staff can be trained through program-wide initiatives to identify caregivers at high risk of significant distress. Further, trained clinical staff can intervene with individualized assessment, education, advocacy, and bereavement support [21]. The ten-year update on this program was recently published. From January 2017 to December 2023, 1544 individual neuro-oncology caregivers participated. The caregiver population was more diverse than previous studies. Approximately 66% of participants were female, 65% spouses, and the majority aged 41–64. Most caregivers were caring for a patient with GBM (59.6%). Recruited caregivers were tracked into one of the following three groups: *GBM Outreach*, *New to Clinic* or *Referrals*. At each meaningful clinical interaction, caregivers were screened with a standard, discrete list of *Concerns* and for care coordination needs using “Care Coordination Notes” within the medical record. Interventions deployed for *Concerns* were similarly tracked in the medical record under “Care Coordination.” The most common concerns expressed by all groups were *Information*, *Emotional* and *Practical*. Caregivers expressed *Concerns* most often in the *Referral* group. Interventions were completed for 84% of caregivers. This remarkable program demonstrates a dedicated neuro-oncology caregiver program is feasible, practical, and helps to rapidly address the support caregivers require during their loved one’s disease course [51].

### 5.2. Psychotherapy and Caregiver Mastery

Psychotherapy and interventions targeted to improve caregiver mastery, or the ability to cope with the demands of care, may offer benefit to reduce caregiver distress; however, the evidence is mixed and limited in GBM. Discussed in greater detail in Section 4.4, a randomized trial of 56 HGG dyads tested six psychologist-led sessions combining psychoeducation and cognitive-behavioral strategies. The authors found that the intervention helped maintain caregiver quality of life and improved mastery over eight months (Table 1) [15].

Discussed briefly in Section 4.4 and in further detail here, an observational analysis of 88 GBM dyads found higher baseline caregiver mastery was associated with longer patient survival after adjustment for measured patient and treatment factors. Specifically, dyads were recruited within three months of patient diagnosis. Only 73% of dyads approached agreed to participate in the study, citing most often lack of interest and feeling overwhelmed. Assessments were performed at 3, 4, 8, and 12 months. The authors used the baseline caregiver assessment as a “potential predictor of patients time to death.” Caregiver anxiety, depression, burden, and mastery were assessed. The authors found that high caregiver mastery significantly extended survival. Importantly, the finding may be confounded by variables not explicitly controlled for in the study, including patient function, disease severity (i.e., MGMT status), patient and caregiver resources, or relationship factors [22]. These findings suggest caregiver mastery may improve survival if caregivers are better able to engage and participate in daily care, appointments, and patient advocacy.

One strategy to support caregiver mastery has been through targeting existential distress rather than daily task-based preparedness. One such example for targeting distress is the “Meaning-Centered Psychotherapy for Cancer Caregivers” program. In a pilot randomized trial of 60 GBM caregivers, seven sessions improved personal meaning, benefit finding, depressive symptoms, and spiritual well-being relative to enhanced usual care [12]. Similarly, across nine GBM caregivers, qualitative interviews suggested the psychotherapy intervention validated the caregiving experience, helped participants place caregiving within a broader identity, and supported agency [52]. Taken together, these studies suggest that quality clinical information alone is unlikely to resolve existential suffering; however, when paired with psychotherapy, caregivers may experience benefit.

### 5.3. Early Palliative and Coordinated Care

Guidelines recommend early palliative-care integration for patients with advanced cancer, including GBM. A large multi-center randomized trial conducted in Germany hypothesized that early palliative care would improve patient QoL and reduce caregiver burden. EPCOG enrolled patients with either newly diagnosed or recurrent GBM, and if available, their caregiver. Patients had to have good performance status and a command of the German language. Patients were excluded for any ongoing psychiatric care or substance use disorder. Patients were randomized to integrated palliative care over the next 12 months or standard of care. In this study, male and female caregivers were more evenly represented (41.8% and 58.2%, respectively). Similar to other cohorts, most caregivers were spouses. The primary objective was to understand how early palliative care involvement affected patient QoL. Secondary objectives included assessment of patients’ needs, psychological health, cognitive outcomes, caregiver burden and overall patient survival. The authors found that with early palliative care, patients experienced improved moods. QoL outcomes favored the intervention when the analysis adjusted for time to death. Caregivers did not appear to benefit from early palliative care in QoL or mood domains [14]. Interpretation of the findings is complicated by a survival imbalance favoring the control group.

Another group has explored how to design a supportive-care intervention focused on dyad psychological health. EMPOWER GBM, a small single-institution pilot study, was a single-arm trial designed to improve GBM dyad QoL and psychological outcomes through targeted, coordinated 45–60 min sessions focused on teaching self-care skills and communication strategies and improving the dyad relationship (Table 1). Importantly, dyads were self-recruited through the Glioblastoma Foundation. Four patients and seven caregivers participated. All caregivers were female. Over six structured interviews, dyads were coached on strategies to manage care and symptoms as a team. Caregivers reported satisfaction with the program and improvement in psychological health. The authors conclude the intervention is feasible, but further work is needed to include more diversity in the dyad population and to understand the true efficacy of the intervention [13]. Similarly, a residential rehabilitative-palliative program over 4 days for GBM dyads focused on paired skills for symptom management, and distress tolerance likewise received high satisfaction ratings [53].

## 6. The Neglected Physical Health of the Caregiver

Most neuro-oncology caregiver interventions prioritize information or care coordination. Far fewer directly address the caregiver’s physical health. Physical health of the caregiver is often significantly impacted. Daily caregiving can disrupt sleep cycles, exercise, eating habits, and personal preventative care appointments. To this point, several qualitative studies describe stress and self-care repeatedly subordinated to patient needs [29,36]. Sleep disturbance, in particular, has been a focus of multiple studies in patients with PBTs. One systematic review found that out of 34 studies examining sleep in patients with PBTs, only one included metrics on caregiver sleep [54].

Similarly to sleep, exercise is often challenging to achieve for patients and caregivers. As discussed above, many prior interventions to improve caregiver distress have focused predominantly on education, readiness, and psychotherapy; however, across the caregiving spectrum, many groups have found that exercise improves caregiver quality of life and psychologic resiliency. In the RECHARGE randomized trial of 77 caregivers of patients with cancer, a 12-week aerobic and resistance-program improved mental health, self-reported exercise, and six-minute walk performance. Most caregivers enrolled had loved ones with breast, prostate, colorectal, or hematological cancers. Small-to-medium effects across several physical and psychological outcomes were also reported [55]. Caregivers of patients with PBTs were not specifically excluded; however, it is difficult to estimate how many were enrolled as neurological cancer fell in the “Others” category. Because the cohort was not restricted to neuro-oncology, transferability is uncertain.

A 2021 systematic review found that most patients with PBTs were insufficiently active. Further, exercise appeared safe and feasible in this population. Possible benefits of aerobic exercise included reduced symptoms, improved aerobic capacity, leaner body composition, and increased physical activity; however, the overall evidence was weak [56]. Similarly, a later review of mind–body, cognitive-behavioral, and physical-activity interventions in a broad group of patients with PBTs described promising but underpowered evidence for mood, quality of life, and physical function [57]. Across multiple studies, patients with PBT report a preference for flexible and individualized programs over generic instructions. Patients frequently identify fatigue, cognitive impairment, treatment effects, and external barriers as major obstacles [58]. An important caveat to these studies is that they were performed in patients with PBTs, irrespective of subtype. Compared to all comers with PBTs, patients with GBM may experience greater symptomatic barriers to physical activity. The intensive treatment and shortened disease course likely present significant challenges to engage in exercise comparatively for GBM patient–caregiver dyads.

## 7. Structured Exercise: Dyadic Opportunity or Additional Caregiving Demand

The literature for dyad exercise in neuro-oncology is best encapsulated by a 2026 scoping review that screened 1126 records and included eight studies of PBT dyads, including four yoga-based interventions, one ski-based intervention, and three aerobic or resistance-training studies [59]. A complementary scoping review identified 11 dyadic psychosocial publications spanning yoga, meditation, psychoeducation, cognitive-behavioral approaches, dignity therapy, and communication interventions [60].

GBM dyads may be receptive to incorporating exercise into the treatment plan. Using semi-structured interviews, a pilot study of 19 patients with HGG and 15 caregivers investigated dyads’ perspectives on participation in interventional exercise during chemoradiation. The authors found that most dyads expressed positive associations around exercise [61].

To date, the most well-developed glioma exercise program is dyadic yoga during radiotherapy with mutual support and interaction. In an initial pilot randomized trial of 20 dyads, adherence and retention benchmarks were met. Patients demonstrated promising improvements in symptom severity, symptom interference, depressive symptoms, and mental quality of life. Further, caregivers demonstrated promising improvements in depressive symptoms, fatigue, and mental quality of life [19]. The sample was too small for definitive efficacy claims, but the study showed that a structured patient–caregiver intervention could be delivered during intensive treatment.

A later three-arm trial directly challenged the assumption that dyadic delivery is always better. Sixty-seven dyads were randomized to dyadic yoga, caregiver-only yoga, or usual care. Attendance was higher for dyadic yoga, but caregivers reported greater subjective benefit from caregiver-only yoga than dyadic yoga (*p* < 0.01). Specifically, authors observed moderate effect-size estimates favoring caregiver-only yoga improved mental quality of life, financial burden, caregiving esteem, and caregiving-related health decline compared to usual care [20]. Dyadic exercise may become less beneficial for the caregiver when the patient has neurologic deficits affecting strength, mobility and balance. As a result, dyadic exercise may increase caregivers’ hypervigilance while trying to prevent falls and encourage safety during exercise. Exercising together can create teamwork and share meaning, but it may also intensify the caregiving role, add further coordination work, and remove private restorative time. Of note, these findings were observed in a yoga-based intervention, which heavily emphasizes mindfulness, and they may not extrapolate to other exercise interventions such as structured functional, strength, or aerobic training where dyadic participation may foster shared effort and mutual accountability rather than competing for restorative space. Further studies are needed in this active area of research.

Most solid tumors do not cause focal neurologic defects impacting balance or safety. As a result, caregivers of other cancer types may have greater comfort with dyadic exercise. To this effect, in other solid tumor types, the evidence suggests dyadic exercise likely benefits both patient and caregiver. In the Exercising Together trial, 64 prostate cancer survivor–spouse couples were randomized to six months of partnered strength training or usual care. The program had high retention and improved physical outcomes in both survivors and spouses [62]. Similarly, a systematic review of caregiver–care recipient exercise dyads concluded that caregivers could benefit when exercising together, but also found psychosocial benefits when caregivers received separate support or respite. No single delivery mode was universally optimal across dyads [63].

Exercise during radiotherapy has been shown across multiple trials to improve radiotherapy-associated fatigue, QOL, anxiety, and depression; however, the evidence regarding the impact of exercise during radiotherapy treatments for PBT is mixed and has shown improved strength preservation, attention, and decreased sleep disturbance compared to controls but not a significant improvement in fatigue or other radiotherapy-related symptoms [64,65,66,67,68].

In GBM, many questions around the different ways dyadic exercise could benefit caregivers and patients remain unanswered. It is unclear how likely the findings of dyadic exercise in other tumor types will generalize to GBM dyads. Focused efforts are required to determine if dyadic or parallel exercise offers the most benefit for caregivers and patients. Further, how best to structure and deliver exercise for dyads, if proven more efficacious, requires further investigation.

## 8. An Adaptive Model of GBM Caregiver Support

Caregivers are indispensable participants in GBM care and experience substantial psychological, practical, relational, and physical consequences. The literature supports a stepwise, adaptive model to improve GBM caregiver well-being that prioritizes caregiver education, caregiver psychosocial supports, maintenance of caregiver physical health, and paired dyad activities designed specifically to build mental and physical reserve together (Figure 1). The caregiving role is dynamic and undergoes frequent revision of responsibilities. As such, caregivers should be evaluated and screened routinely for well-being, with appropriate resources allocated as needed. Needs assessment could be reasonably performed at diagnosis, reassessed after surgery, during chemoradiation, surveillance transitions, with functional decline or progression, when goals of care change, and after death. Targeted services identified during needs assessment could be deployed through a formal neuro-oncology referral system. Required targeted services include psychotherapy, social work and financial navigation, rehabilitation and home health services, palliative-care consultation, and advance-care planning. Beyond targeted referrals, health-promoting support should include the caregiver’s own sleep, medical care, and physical activity. Delivery of exercise as dyad or caregiver-only should be tailored to family preference. Given the complex and rapid course of GBM, feasibility of dyad-based exercise may shift dramatically over the disease course. Finally, caregivers express need for anticipatory guidance around end-of-life and bereavement care. Caregivers may benefit from explicit plans detailing how to identify end-of-life, whom to call, and what to do at the time of death. Caregiver follow-up after death should include screening for grief, depression, anxiety, trauma symptoms, family disruption, and practical needs, with clear referral options. Substantial work remains to establish best practices and evidence-based interventions to reduce GBM caregiver distress. Future work should prioritize interventions that readily improve both mental and physical health of the dyad, ultimately improving patient survival and caregiver well-being.

## 9. Limitations and Equity/Implementation Considerations

The literature examining supportive-care interventions for GBM caregivers has several limitations. Most interventional studies involve small samples, heterogeneous populations, and variable outcome measures, limiting the generalizability of their findings. Further, many studies enroll caregivers of patients with high-grade gliomas or other primary brain tumors rather than GBM exclusively. A notable limitation is the demographic homogeneity of enrolled caregivers. Several studies have predominantly recruited White, female, and well-educated caregivers with relatively high socioeconomic status [20,41]. For example, the Care-IS trial required English-language proficiency, while caregivers enrolled in EMPOWER GBM were exclusively female [13,16]. Similarly, the three-arm trial comparing dyadic and caregiver-only yoga predominantly enrolled female and non-Hispanic White caregivers [20]. Consequently, the applicability of these interventions to more diverse caregiver populations remains uncertain.

Implementing supportive-care interventions across diverse populations presents additional challenges. Caregivers facing financial insecurity, employment obligations, transportation difficulties, language barriers, or limited access to supportive-care services may encounter significant obstacles to participation. These barriers may be particularly relevant to structured exercise interventions requiring regular attendance, supervision, or specialized equipment. Additionally, the neurologic deficits and functional limitations associated with GBM may further restrict the feasibility of dyadic exercise. Caregiver-only interventions may offer greater flexibility and opportunities for independent respite, although the optimal approach likely varies according to individual circumstances and preferences.

Future supportive-care programs should prioritize accessibility and flexibility through multilingual resources, culturally responsive interventions, remote or community-based delivery options, and individualized scheduling. Importantly, remote interventions should not be assumed to eliminate access barriers, particularly for caregivers with limited digital resources. Future trials should intentionally recruit underrepresented caregiver populations, report demographic and socioeconomic characteristics, and evaluate whether intervention feasibility and effectiveness differ across these groups. Addressing these considerations will be essential to developing supportive-care models that are both effective and generalizable to the broader GBM caregiver population.

## 10. Discussion

Caregivers of GBM patients frequently navigate cognitive, behavioral, and physical declines in the patient while enduring psychological strain, physical exhaustion, and financial burnout. Addressing these complex challenges requires clinical trial strategies and institutional support that explicitly recognize the patient–caregiver dyad as the primary recipient of intervention. Future clinical trials must prioritize robustly designed dyadic interventions by engaging patients, caregivers, community advocacy groups, and key stakeholders early and continuously as equal partners. Significant effort has been made to standardize neuro-oncology trials involving caregivers, with new recommendations from RANO-cares working group [69]. A collaborative approach is essential for designing pragmatic, real-world trials that can successfully recruit and retain the full patient–caregiver dyad. Rather than isolating caregiver supportive care from active oncologic management, future trial protocols should co-evaluate patient and caregiver outcomes as an interdependent team. Translating this research into scalable, routine practice will require strong institutional commitment and health system infrastructure.

Dedicated clinical models, such as the UCSF Neuro-Oncology Gordon Murray Caregiver Program, demonstrate the immense value of integrating systematic caregiver assessments, dedicated social work, nurse navigation, and peer support directly into neuro-oncology workflows (Table 1) [21]. A subsequent 10-year report from the program described 1544 caregivers with the program encounters; informational (42%), emotional (25%), and practical (24%) concerns were most common, and 84% received at least one intervention. The Gordon Murray Caregiver Program establishes that a dedicated neuro-oncology caregiver program is not only practical, but necessary to urgently address unmet caregiver needs [51]. Future multicenter initiatives should build upon this foundation by utilizing an adaptive model for caregiver intake, implementing automated distress screening, and establishing streamlined referral pathways to targeted supportive-care services. In parallel, building prospective, longitudinal patient–caregiver registries represents a high-impact opportunity for the field. Existing neuro-oncology registries focus almost exclusively on patient survival, progression, and treatment toxicity, leaving caregiver health, cognitive fatigue, and financial strain unmonitored. Capturing these longitudinal data will be essential for benchmarking care standards, demonstrating the cost-effectiveness of dyadic interventions to key stakeholders, and ultimately improving outcomes for dyads navigating glioblastoma.

## 11. Conclusions

Glioblastoma imposes a profound, interdependent toll on patients and their caregivers due to rapid neurological decline and aggressive oncologic progression. Beyond their baseline relationship, caregivers are frequently forced to step into additional complex roles, serving as home nurses for outpatient chemotherapy administration, health system navigators, social workers, and primary emotional anchors. This deep interdependence highlights the critical need to shift from traditional patient-centric models toward a formal dyadic paradigm in neuro-oncology supportive care. Because dyadic needs evolve dynamically (from diagnosis and active treatment through surveillance, progression, and bereavement), supportive care must be adaptive. This requires targeted initiatives, including nurse-led navigation, structured education, anticipatory guidance, and dedicated clinical trials designed to improve both physical and psychological well-being for the dyad at different stages in the disease course. To establish an optimal supportive-care framework in glioblastoma, future trials must look beyond standard psychological metrics and patient survival to evaluate implementation feasibility, clinical trial enrollment, and healthcare utilization savings.

## Figures and Tables

**Table 2:** Caregiver responsibilities, unmet needs, and suggested supportive responses across the high-grade glioma trajectory.

Disease Phase	Patient Symptoms	Caregiver Responsibilities	Caregiver Needs	Supportive Response	
Diagnosis, surgery, and discharge	New neurologic deficits; postoperative fatigue or delirium; uncertain prognosis; impaired recall or decisional capacity	Advocacy; discharge preparation; medication, wound, and seizure management; transportation; rehabilitation coordination	Staged information; practical skills; named clinical contact; emergency guidance; role preparation	Caregiver identification; repeated teach-back; written action plans; early navigation; rehabilitation referral	[8,24,25,26]
Chemoradiation and early outpatient care	Fatigue; nausea; steroid effects; infection risk; seizures; frequent appointments	Oral treatment administration; symptom surveillance; logistics; work and household management	Treatment-specific education; coping support; financial and employment resources; respite	Needs screening; nurse follow-up; social work; home-based or telehealth support; symptom-management training	[16,27,28,29]
Surveillance or relative stability	Persistent cognitive, behavioral, or physical deficits; scan-related uncertainty	Long-term supervision; adapting family roles; sustaining routines and safety	Psychological support; social reconnection; self-care; anticipatory guidance	Periodic reassessment; peer or psychological support; caregiver health promotion; exercise options	[30,31,32,33,34]
Progression and increasing dependence	Declining mobility, cognition, communication, or independence; uncertain treatment benefit	Personal care; equipment and home-safety decisions; surrogate decision-making; care coordination	Clear prognostic communication; palliative and rehabilitation guidance; practical home support	Early palliative care; advance-care planning; home health; equipment; caregiver-only and dyadic services	[15,22,23,35,36]
End of life	Somnolence; dysphagia; agitation; seizures; loss of communication and mobility	Intensive home care; symptom observation; surrogate decisions; preparation for death	Anticipatory guidance; 24 h contact plan; hospice education; emotional and spiritual support	Hospice or palliative services; explicit dying-process education; crisis plan; respite; family support	[14,37,38,39]
Bereavement	Patient death and termination of clinical contact	Practical arrangements; family reorganization; grief management	Grief, trauma, depression, anxiety, financial and social support	Proactive follow-up; bereavement screening; referral; peer and family services	[14,21,36,40]

## Data Availability

No new data were created or analyzed in this study. Data sharing is not applicable to this article.

## Abbreviations

The following abbreviations are used in this manuscript:

GBM	Glioblastoma
TMZ	Temozolomide
HGG	High-Grade Glioma
PBT	Primary Brain Tumor
QoL	Quality of Life
ADLs	Activities of Daily Living
RCT	Randomized Controlled Trial
